# Mast cells are at the interface between the external environment and the inner organism

**DOI:** 10.3389/fmed.2023.1332047

**Published:** 2024-01-04

**Authors:** Domenico Ribatti

**Affiliations:** Department of Translational Biomedicine and Neuroscience, University of Bari Medical School, Bari, Italy

**Keywords:** mast cells, gastrointestinal tract, lung, skin, barrier system

## Abstract

Mast cells localized at the level of the mucosal barrier in the skin, lung, and gastrointestinal tract, intervene in the modulation of the function of the epithelial cells and are involved in innate and adaptive defensive responses. In this context, mast cells intervene in the recognition and clearance of microbial pathogens. This mini-review article discusses the role of mast cells in these barrier systems.

## Introduction

In rodents, mast cells are classified into mucosal mast cells (MMCs) or connective tissue mast cells (CTMCs) (1). MMCs are localized between epithelial cells in the mucosa of the lung and gastrointestinal tract. In their cytoplasmatic granules, chondroitin sulfate and a few amounts of histamine and carboxypeptidase, are present (2), whereas CTMCs are localized in the intestinal submucosa, skin, peritoneal cavity, and generally occupy a perivascular position surrounding blood vessels. In their granules heparin and large amounts of histamine and carboxypeptidase are present (3).

Another possibility to classify mast cells in humans is founded on the presence in their granules of high levels of tryptase but little or no chymase (MC_T_) in intestinal and pulmonary mucosa, predominantly found at mucosal sites, resembling rodent MMCs, or mast cells containing chymase, and little or no tryptase (MC_C_), and finally, mast cells containing tryptase, chymase, and carboxypeptidase (MC_TC_), resembling rodent CTMCs, predominantly found in the skin, lymph nodes, and lung and gut submucosa (4, 5). In the human small intestine, MC_T_ constitutes ∼98% of all mast cells in the mucosa, while ∼13% of MCs in the submucosa are MC_T_ (6). The protease content of mast cells is strictly correlated with heterogeneous cytokines and receptor expression.

More recently, Tauber et al. (7) using *in silico* investigation, have analyzed single-cell profiles of mast cells from different organs in mice and humans. In these latter, they identified seven mast cell subsets distributed in 12 organs with different transcriptomic core signatures. MC1 is preferentially localized in the bladder, MC2 in the lung, and MC4, MC6, and MC7 in the skin. MC3 and MC5 are localized in different organs, but not in the skin.

The contribution of mast cells to mucosal barrier control is mediated through the modulation of epithelial function and innate and adaptive immunological responses. The skin, lung, and gastrointestinal tract are potential portals of entry for foreign agents and are involved in the recognition and clearance of microbial pathogens (8), through the modulation of defense functions in these strategically important sites (9, 10). Activation of mast cells in the skin, gastrointestinal tract, and airways mediates the release of several mediators, responsible for different symptoms (Table 1).

**Table 1:** Different symptoms induced by the release of mast cell mediators in the skin, gastrointestinal tract, and airways.

**Skin**
Flushing
Pruritus
Urticaria
Angioedema
**Gastrointestinal tract**
Abdominal pain
Diarrhea
Esophageal reflux
Nausea
Vomiting
**Airways**
Nasal congestion
Nasal pruritus
Bronchospasm
Swelling
Wheezing

## Mast cells in the skin

The most important skin function is to protect the host from invasion, using physical barriers and a complex network of resident immune cells, including macrophages, T and B lymphocytes, mast cells, neutrophils, eosinophils, and Langerhans cells, and non-immune cells. Skin mast cells are regulated by the skin microbiota extending from the surface to the dermis and dermal adipose tissue. In germ-free mice, few mast cells are recognizable in the dermis, and the intradermal injection in these mice of staphylococcal-derived lipoteichoic acid is responsible for the induction of the expression of stem cell factor (SCF) in keratinocytes, which induces the rescue of the dermal mast cells (11).

Skin mast cells are formed by three distinct cell populations, derived from the yolk sac and the aorta–gonad–mesonephros–derived hematopoietic stem cells in pre-natal life, and bone marrow in post-natal life (12, 13). SCF is secreted by different cells in the skin, and promotes skin mast cell differentiation (11, 14–16).

The number of skin mast cells involved in immune response and host defense (17) varies between 5,120 and 9,472 per mm^3^ in physiological conditions, to 260,000/380,000 per mm^3^ in pathological conditions, such as mastocytosis (18). Moreover, their number is greatest in the skin’s superficial layers as compared with the deep layers (19), and are localized around hair follicles (20), sebaceous and sweat glands, near small blood vessels, and near nerve fibers positive to vasoactive intestinal peptide (VIP), which suppresses mast cell degranulation (21). Small mast cells are found in the subepithelial layer of the skin, and their size and granule content gradually increase in the deep layers.

Skin mast cells are a source of cytokines, chemokines, and growth factors (22, 23). Mast cells induce skin fibroblast proliferation via interleukin (IL)-4 (24, 25), IL-13 (26), vascular endothelial growth factor (VEGF), and basic fibroblast growth factor (bFGF) (27–29). Moreover, mast cells contribute and interact with keratinocytes (30, 31), which, in turn, produce SCF (11, 32). Mast cells synthesize and release keratinocyte growth factor (KGF) (33) and platelet-activating factor (PAF) (31, 34) that activate keratinocytes, and in the meantime, they release histamine, heparin, which inhibit keratinocyte proliferation, controlling epidermal regeneration (35, 36). Finally, tryptase and chymase stimulate fibroblast, inhibit keratinocyte proliferation (37), and regulate epidermal differentiation complex genes, modulating epidermal barrier integrity (38).

Mast cells are involved in many different pathological conditions, including acute and chronic contact hypersensitivity, psoriasis, wound healing, and fibrosis. In acute contact hypersensitivity, mast cell-derived tumor necrosis factor-alpha (TNFα) enhances skin dendritic cell migration to draining lymph nodes, inducing Th-cell expansion in the sensitization phase of contact hypersensitivity (39). In chronic contact hypersensitivity, crosslinked hapten/hapten-specific IgG complexes stimulate mast cells to synthesize IL-10, which, in turn, suppresses the inflammatory reaction (40). In psoriasis, mast cells synthesize TNFα, which promotes dendritic cell migration from the skin to draining lymph nodes (41). In wound healing and fibrosis, mast cells degranulate and release mediators enhances vascular permeability, and recruit inflammatory cells in injured sites. Moreover, mast cell mediators activate fibroblasts to release collagen-promoting fibrosis (42, 43).

## Mast cells in the gastrointestinal tract

The intestinal mucosa is lined with enterocytes, separating the lumen from the internal milieu, avoiding the passage of harmful substances, and allowing nutrient and electrolyte absorption. The gut may be considered as a barrier with a defensive function exerted through immune responses, and the acquisition of tolerance against food antigens and the microbiota. The intestinal leakage allows the bacteria to enter the bloodstream, activate pattern recognition receptors, and trigger an immune response (44).

Mast cells are present in all layers of the gastrointestinal tract; their number is higher in the mucosa’s lamina propria of the duodenum and colon, as compared to the submucosa (5, 45). Mast cells are preferentially localized next to nerve terminals of the lamina propria, and are activated by substance P, which stimulates mast cells to release inflammatory mediators, including serotonin and proteases, and pro-inflammatory cytokines.

Intestinal mast cells perform intervene in the regulation of ion and water secretion and permeability, blood flow, coagulation, vascular permeability, wound healing, fibrosis, neuro-immune interactions, peristalsis, pain perception, bacterial, viral, and parasitic infections, and innate and adaptive immunity reactions (4, 46–48). All these activities are exerted by intestinal mast cells in physiological conditions and explain their involvement in many pathological conditions including allergic disorders, gastrointestinal infections, chronic inflammatory diseases, and colon cancer (49).

An increased number of mast cells in inflamed segments of the bowel compared with the non-inflamed segments has been demonstrated in patients with chronic inflammatory bowel disease, including ulcerative colitis and Crohn’s disease. An impaired balance between pro- and anti-inflammatory mediators released by mast cells causes excessive inflammatory reactions and subsequently symptoms such as abdominal cramps, diarrhea, and rectal bleeding (50). Mast cells are involved in the inflammatory process in celiac disease. Intestinal bioptic specimens of patients with celiac disease show a correlation between mast cell density and disease severity (51). Gastrointestinal mastocytosis is characterized by an accumulation of mast cells in the gastrointestinal tissue, which may occur during systemic mastocytosis involving other organs such as bone marrow, liver, and skin. Mast-cell infiltration can result in organ dysfunction in aggressive systemic mastocytosis, and gastrointestinal symptoms, including abdominal pain and diarrhea, are present in 60–80% of cases (52). The term mastocytic enterocolitis was coined to describe an increase in mucosal mast cells in patients with chronic diarrhea (53).

## Mast cells in the airways

Each of the MC_T_ and MC_TC_ populations can be further differentiated based on their size, shape, and molecular expression profiles in different lung compartments (54, 55). More than 90% of the mast cells located in bronchi, bronchioles, and alveolar walls are MC_T_, whereas MC_TC_ is localized in the subepithelial regions and the connective tissue (56). The higher number of mast cells are localized in the alveoli, where they are involved in the homeostatic regulation of blood flow and act as immune modulators and pro-fibrotic cells.

Bronchial MC_T_ expresses more histidine decarboxylase than alveolar MC_T_. In contrast, in both MC_TC_ and MC_T_, the high-affinity receptor for IgE (FcεRI) is expressed in conducting airways but absent in alveolar parenchyma (54). IgE-mediated mast cell degranulation is an established hallmark of allergic respiratory conditions, and none of the mast cell effector functions executed in allergy and asthma take place in the alveolar parenchyma.

In addition to the evidence presented for the role of mast cells in the pathophysiology of asthma (57), there is growing evidence that mast cells may also play roles in other diseases of the airways, such as chronic obstructive pulmonary disease (COPD) (58). Mast cell numbers in the lungs of patients with fibrotic disease are increased compared to control subjects and correlate with the severity of fibrosis (59). Patients with idiopathic pulmonary arterial hypertension have marked infiltration of Kit^+^ mast cells near remodeled pulmonary arteries with a predominant perivascular distribution (60). Initial stages of viral rhinitis are characterized by the high presence of mast cells which, together with other immune cells such as T and B lymphocytes, start the inflammatory cascade in the upper airways (61). Histological examination of the lungs of patients who died from SARS-CoV-2 infection revealed extensive infiltration of mast cells in the interstitium and alveoli (62).

## Concluding remarks

Mast cells are involved in the first line of defense against foreign pathogens, producing antimicrobial peptides and releasing different pre-formed and newly synthesized mediators. Mast cells exert protection against systemic or localized bacterial infections in the skin, gastrointestinal tract, and airways by facilitating the clearance of enterobacteria from these tissues.

Mast cells exert this action by engulfing bacteria and secrete inflammatory mediators that, in turn, recruit and activate phagocytes, such as neutrophils through the secretion of TNFα, leukotrienes B4 and C4, and are implicated in the protective response (63). The local increase in mast cell number at the site of infection mediated by the c-kit ligand SCF amplifies mast cell-mediated immune response (64). Mast cells themselves are involved in phagocytosis and the production of nitric oxide, superoxide radicals, and antimicrobial peptides. As concerns viral infection, mast cell number increases during pulmonary viral infections in humans (65), and toll-like receptor 3 expression by human mast cells is implicated in the production of interferon-alpha in response to viral exposure (66).

## Author contributions

DR: Writing – original draft, Writing – review & editing.

## References

[1] Irani AM Schwartz LB. Mast cell heterogeneity. Clin Exp Allergy. (1989) 19:143–55. doi: 10.1111/j.1365-2222.1989.tb02357.x 2473830

[2] Katz HR Stevens RL Austen KF. Heterogeneity of mammalian mast cells differentiated *in vivo* and *in vitro*. J Allergy Clin Immunol. (1985) 76:250–9. doi: 10.1016/0091-6749(85)90638-4, PMID: 2410474

[3] Lowman MA Rees PH Benyon RC Church MK. Human mast cell heterogeneity: histamine release from mast cells dispersed from skin, lung adenoids, tonsils, and colon in response to IgE-dependent and nonimmunologic stimuli. J Allergy Clin Immunol. (1988) 81:590–7. 2450114

[4] Bischoff SC. Role of mast cells in allergic and non-allergic immune responses: comparison of human and murine data. Nat Rev Immunol. (2007) 7:93–104. doi: 10.1038/nri2018, PMID: 17259966

[5] Irani A Schechter N Craig S DeBlois G Schwartz L. Two types of human mast cells that have distinct neutral protease compositions. Proc Natl Acad Sci U S A. (1986) 83:4464–8. doi: 10.1073/pnas.83.12.4464, PMID: 3520574 PMC323754

[6] Reber LL Sibilano R Mukai K Galli SJ. Potential effector and immunoregulatory functions of mast cells in mucosal immunity. Mucosal Immunol. (2015) 8:444–63. doi: 10.1038/mi.2014.131, PMID: 25669149 PMC4739802

[7] Tauber M Basso L Martin J Bostan L Magalhaes Pinto M Thierry GR. Landscape of mast cell populations across organs in mice and humans. J Exp Med. (2023) 220:e20230570. doi: 10.1084/jem.20230570, PMID: 37462672 PMC10354537

[8] Jimenez M Cervantes-García D Córdova-Dávalos LE Pérez-Rodríguez MJ Gonzalez-Espinosa C Salinas E. Responses of mast cells to pathogens: beneficial and detrimental roles. Front Immunol. (2021) 12:685865. doi: 10.3389/fimmu.2021.685865, PMID: 34211473 PMC8240065

[9] Ferguson A Cummins AG Munro GH Gibson S Miller HR. Role of mucosal mast cells in intestinal cell-mediated immunity. Ann Allergy. (1987) 59:40–3. 3479922

[10] Fonseca E Solis J. Mast cells in the skin: progressive systemic sclerosis and the toxic oil syndrome. Ann Int Med. (1985) 102:864–5. doi: 10.7326/0003-4819-102-6-864_2, PMID: 3994202

[11] Wang Z Mascarenhas N Eckmann L Miyamoto Y Sun X Kawakami T. Skin microbiome promotes mast cell maturation by triggering stem cell factor production in keratinocytes. J Allergy Clin Immunol. (2017) 139:1205–1216.e6. doi: 10.1016/j.jaci.2016.09.019, PMID: 27746235 PMC5385284

[12] Gentek R Ghigo C Hoeffel G Bulle MJ Msallam R Gautier G. Hemogenic endothelial fate mapping reveals dual developmental origin of mast cells. Immunity. (2018) 48:1160–1171.e5. doi: 10.1016/j.immuni.2018.04.025, PMID: 29858009

[13] Li Z Liu S Xu J Zhang X Han D Liu J. Adult connective tissue-resident mast cells originate from late erythro-myeloid progenitors. Immunity. (2018) 49:640–653.e5. doi: 10.1016/j.immuni.2018.09.023, PMID: 30332630

[14] Cicek F Cekic S Kilic SS. Infliximab therapy in an infant with Netherton syndrome. Pediatr Dermatol. (2021) 38:714–6. doi: 10.1111/pde.14590, PMID: 33890311

[15] Kambe N Kambe M Kochan JP Schwartz LB. Human skin derived mast cells can proliferate while retaining their characteristic functional and protease phenotypes. Blood. (2001) 97:2045–52. doi: 10.1182/blood.V97.7.2045, PMID: 11264170

[16] Salducci M André N Guéré C Martin M Fitoussi R Vié KM. Factors secreted by irradiated aged fibroblasts induce solar lentigo in pigmented reconstructed epidermis. Pigment Cell Melanoma Res. (2014) 2014:502–4. doi: 10.1111/pcmr.12234 24533682

[17] Henz BM Mauer M Lippert U Worm M Babina M. Mast cells as initiators of immunity and host defense. Exp Dermatol. (2001) 10:1–10. doi: 10.1034/j.1600-0625.2001.100101.x 11168574

[18] Mikhail GR Milinska A. Mast cell population in human skin. J Invest Dermatol. (1964) 43:249–54. doi: 10.1038/jid.1964.152 14220232

[19] Weber A Knop J Maurer M. Pattern analysis of human cutaneous mast cell populations by total body surface mapping. Br J Dermatol. (2003) 148:224–8. doi: 10.1046/j.1365-2133.2003.05090.x, PMID: 12588371

[20] Ito N Sugawara K Bodó E Takigawa M van Beek N Ito T. Corticotropin-releasing hormone stimulates the in situ generation of mast cells from precursors in the human hair follicle mesenchyme. J Invest Dermatol. (2010) 130:995–1004. doi: 10.1038/jid.2009.387 20043013

[21] Pilkington SM Barron MJ Watson REB Grifths CEM Bulfone-Paus S. Aged human skin accumulates mast cells with altered functionality that localize to macrophages and vasoactive intestinal peptide-positive nerve fibers. Br J Dermatol. (2019) 180:849–58. doi: 10.1111/bjd.17268, PMID: 30291626 PMC6619242

[22] Mukai K Tsai M Saito H Galli SJ. Mast cells as sources of cytokines, chemokines, and growth factors. Immunol Rev. (2018) 282:121–50. doi: 10.1111/imr.12634, PMID: 29431212 PMC5813811

[23] Tan SY Roediger B Weninger W. The role of chemokines in cutaneous immunosurveillance. Immunol Cell Biol. (2015) 93:337–46. doi: 10.1038/icb.2015.16, PMID: 25776847

[24] Richter A Puddicombe SM Lordan JL Bucchieri F Wilson SJ Djukanovic R. The contribution of interleukin (IL)-4 and IL-13 to the epithelial-mesenchymal trophic unit in asthma. Am J Respir Cell Mol Biol. (2001) 25:385–91. doi: 10.1165/ajrcmb.25.3.4437, PMID: 11588018

[25] Trautmann A Toksoy A Engelhardt E Bröcker EB Gillitzer R. Mast cell involvement in normal human skin wound healing: expression of monocyte chemoattractant protein-1 is correlated with recruitment of mast cells which synthesize interleukin-4 *in vivo*. J Pathol. (2000) 190:100–6. doi: 10.1002/(SICI)1096-9896(200001)190:1<100::AID-PATH496>3.0.CO;2-Q, PMID: 10640999

[26] Brown Lobbins ML Shivakumar BR Postlethwaite AE Hasty KA. Chronic exposure of interleukin-13 suppress the induction of matrix metalloproteinase-1 by tumour necrosis factor α in normal and scleroderma dermal fibroblasts through protein kinase B/Akt. Clin Exp Immunol. (2018) 191:84–95. doi: 10.1111/cei.13045, PMID: 28884475 PMC5721252

[27] Komi DEA Khomtchouk K Santa Maria PL. A review of the contribution of mast cells in wound healing: involved molecular and cellular mechanisms. Clin Rev Allergy Immunol. (2020) 58:298–312. doi: 10.1007/s12016-019-08729-w 30729428

[28] Qu Z Huang X Ahmadi P Stenberg P Liebler JM Le AC. Synthesis of basic fibroblast growth factor by murine mast cells. Regulation by transforming growth factor beta, tumor necrosis factor alpha, and stem cell factor. Int Arch Allergy Immunol. (1998) 115:47–54. doi: 10.1159/000023829, PMID: 9430495

[29] Tellechea A Leal EC Kafanas A Auster ME Kuchibhotla S Ostrovsky Y. Mast cells regulate wound healing in diabetes. Diabetes. (2016) 65:2006–19. doi: 10.2337/db15-0340, PMID: 27207516 PMC4915574

[30] Nguyen AV Soulika AM. The dynamics of the skin’s immune system. Int J Mol Sci. (2019) 20:1811. doi: 10.3390/ijms20081811, PMID: 31013709 PMC6515324

[31] Varricchi G Rossi FW Galdiero MR Granata F Criscuolo G Spadaro G. Physiological roles of mast cells: collegium Internationale Allergologicum update 2019. Int Arch Allergy Immunol. (2019) 179:247–61. doi: 10.1159/000500088 31137021

[32] Cho KA Park M Kim YH Woo SY. Th17 cell-mediated immune responses promote mast cell proliferation by triggering stem cell factor in keratinocytes. Biochem Biophys Res Commun. (2017) 487:856–61. doi: 10.1016/j.bbrc.2017.04.141, PMID: 28456630

[33] Cho KA Kim HJ Kim YH Park M Woo SY. Dexamethasone promotes keratinocyte proliferation by triggering keratinocyte growth factor in mast cells. Int Arch Allergy Immunol. (2019) 179:53–61. doi: 10.1159/000494624, PMID: 30909282

[34] Feuerherm AJ Jørgensen KM Sommerfelt RM Eidem LE Lægreid A Johansen B. Platelet-activating factor induces proliferation in differentiated keratinocytes. Mol Cell Biochem. (2013) 384:83–94. doi: 10.1007/s11010-013-1784-6, PMID: 23975504

[35] Gschwandtner M Mildner M Mlitz V Gruber F Eckhart L Werfel T. Histamine suppresses epidermal keratinocyte differentiation and impairs skin barrier function in a human skin model. Allergy. (2013) 68:37–47. doi: 10.1111/all.12051, PMID: 23157658 PMC3555427

[36] Huttunen M Hyttinen M Nilsson G Butterfield JH Horsmanheimo M Harvima IT. Inhibition of keratinocyte growth in cell culture and whole skin culture by mast cell mediators. Exp Dermatol. (2001) 10:184–92. doi: 10.1034/j.1600-0625.2001.010003184.x, PMID: 11380614

[37] Artuc M Steckelings UM Grützkau A Smorodchenko A Henz BM. A long-term coculture model for the study of mast cell-keratinocyte interactions. J Investig Dermatol. (2002) 119:411–5. doi: 10.1046/j.1523-1747.2002.01838.x, PMID: 12190864

[38] Sehra S Serezani APM Ocaña JA Travers JB Kaplan MH. Mast cells regulate epidermal barrier function and the development of allergic skin inflammation. J Investig Dermatol. (2016) 136:1429–37. doi: 10.1016/j.jid.2016.03.019, PMID: 27021404 PMC4921316

[39] Gaudenzio N Marichal T Galli SJ Reber LL. Genetic and imaging approaches reveal pro-inflammatory and immunoregulatory roles of mast cells in contact hypersensitivity. Front Immunol. (2018) 9:1275. doi: 10.3389/fimmu.2018.01275, PMID: 29922295 PMC5996070

[40] Grimbaldeston MA Nakae S Kalesnikoff J Tsai M Galli SJ. Mast cell-derived interleukin 10 limits skin pathology in contact dermatitis and chronic irradiation with ultraviolet B. Nat Immunol. (2007) 8:1095–104. doi: 10.1038/ni1503, PMID: 17767162

[41] Heib V Becker M Warger T Rechtsteiner G Tertilt C Klein M. Mast cells are crucial for early inflammation, migration of Langerhans cells, and Ctl responses following topical application of Tlr7 ligand in mice. Blood. (2007) 110:946–53. doi: 10.1182/blood-2006-07-036889, PMID: 17446350

[42] Levi-Schaffer F Kupietzky A. Mast cells enhance migration and proliferation of fibroblasts into an *in vitro* wound. Exp Cell Res. (1990) 188:42–9. doi: 10.1016/0014-4827(90)90275-F, PMID: 2328776

[43] Wulff BC Wilgus TA. Mast cell activity in the healing wound: more than meets the eye? Exp Dermatol. (2013) 22:507–10. doi: 10.1111/exd.12169, PMID: 23802591 PMC3723719

[44] Levy M Kolodziejczyk AA Thaiss CA Elinav E. Dysbiosis and the immune system. Nat Rev Immunol. (2017) 17:219–32. doi: 10.1038/nri.2017.7 28260787

[45] Bischoff SC Wedemeyer J Herrmann A Meier PN Trautwein C Cetin Y. Quantitative assessment of intestinal eosinophils and mast cells in inflammatory bowel disease. Histopathology. (1996) 28:1–13. doi: 10.1046/j.1365-2559.1996.262309.x, PMID: 8838115

[46] Alpers DH Andrews PL Wood JD. Fundamental of gastroenterology. Gut. (1999) 45:6–7. doi: 10.1136/gut.45.1.6, PMID: 10457039 PMC1766686

[47] Bischoff SC. Physiological and pathophysiological functions of intestinal mast cells. Semin Immunopathol. (2009) 31:185–205. doi: 10.1007/s00281-009-0165-4 19533134

[48] Crowe SE Luthra G Perdue M. Mast cell mediated ion transport in intestine from patients with and without inflammatory bowel disease. Gut. (1997) 41:785–92. doi: 10.1136/gut.41.6.785, PMID: 9462211 PMC1891616

[49] Bischoff SC. Mast cells in gastrointestinal disorders. Eur J Pharmacol. (2016) 778:139–45. doi: 10.1016/j.ejphar.2016.02.018 26852959

[50] Nishida Y Murase K Isomoto H Furusu H Mizuta Y Riddell RH. Different distribution of mast cells and macrophages in colonic mucosa of patients with collagenous colitis and inflammatory bowel disease. Hepatogastroenterology. (2002) 49:678–82. PMID: 12063968

[51] Frossi B Tripodo C Guarnotta C Carroccio A De Carli M De Carli S. Mast cells are associated with the onset and progression of celiac disease. J Allergy Clin Immunol. (2017) 139:1266–1274.e1. doi: 10.1016/j.jaci.2016.08.011, PMID: 27619824

[52] Sokol H Georgin-Lavialle S Grandpeix-Guyodo C Canioni D Barete S Dubreuil P. Gastrointestinal involvement and manifestations in systemic mastocytosis. Inflamm Bowel Dis. (2010) 16:1247–53. doi: 10.1002/ibd.21218, PMID: 20162539

[53] Jakate S Demeo M John R Tobin M Keshavarzian A. Mastocytic enterocolitis: increased mucosal mast cells in chronic intractable diarrhea. Arch Pathol Lab Med. (2006) 130:362–7. doi: 10.5858/2006-130-362-MEIMMC, PMID: 16519565

[54] Andersson CK Mori M Bjermer L Löfdahl CG Erjefält JS. Novel site-specific mast cell subpopulations in the human lung. Thorax. (2009) 64:297–305. doi: 10.1136/thx.2008.101683, PMID: 19131451

[55] Bradding P. Human lung mast cell heterogeneity. Thorax. (2009) 64:278–80. doi: 10.1136/thx.2008.106427 19329727

[56] Schwartz LB Bradford TR Irani AM DeBlois G Craig SS. The major enzymes of human mast cell secretory granules. Am Rev Respir Dis. (1987) 135:1186–9. PMID: 3555191 10.1164/arrd.1987.135.5.1186

[57] Bradding P Walls AF Holgate ST. The role of the mast cell in the pathophysiology of asthma. J Allergy Clin Immunol. (2006) 117:1277–84. doi: 10.1016/j.jaci.2006.02.039 16750987

[58] Andersson CK Mori M Bjermer L Lofdahl CG Erjefalt JS. Alterations in lung mast cell populations in patients with chronic obstructive pulmonary disease. Am J Respir Crit Care Med. (2010) 181:206–17. doi: 10.1164/rccm.200906-0932OC 19926870

[59] Pesci A Bertorelli G Gabrielli M Olivieri D. Mast cells in fibrotic lung disorders. Chest. (1993) 103:989–96. doi: 10.1378/chest.103.4.989 8131513

[60] Montani D Perros F Gambaryan N Girerd B Dorfmuller P Price LC. C-kit-positive cells accumulate in remodeled vessels of idiopathic pulmonary arterial hypertension. Am J Respir Crit Care Med. (2011) 184:116–23. doi: 10.1164/rccm.201006-0905OC 21471108

[61] Piliponsky AM Acharya M Shubin NJ. Mast cells in viral, bacterial, and fungal infection immunity. Int J Mol Sci. (2019) 20:2851. doi: 10.3390/ijms20122851, PMID: 31212724 PMC6627964

[62] Conti P Caraffa A Tete G Gallenga CE Ross R Kritas SK. Mast cells activated by SARS-CoV-2 release histamine which increases IL-1 levels causing cytokine storm and inflammatory reaction in COVID-19. J Biol Regul Homeost Agents. (2020) 34:1629–32. doi: 10.23812/20-2EDIT, PMID: 32945158

[63] Marshall JS. Mast cell responses to pathogens. Nat Rev Immunol. (2004) 4:787–99. doi: 10.1038/nri1460 15459670

[64] Maurer M Echtenacher B Hültner L Kollias G Männel DN Langley KE. The c-kit ligand, stem cell factor, can enhance innate immunity through effects on mast cells. J Exp Med. (1998) 188:2343–8. doi: 10.1084/jem.188.12.2343, PMID: 9858520 PMC2212432

[65] Casleman WL Sorkness RL Lemanske RF Jr McAllister PK. Viral bronchiolitis during early life induces increased numbers of bronchiolar mast cells and airway hyperresponsiveness. Am J Pathol. (1990) 137:821–31. 1699421 PMC1877564

[66] Kulka M Alexopoulou L Flavell RA Metcalfe DD. Activation of mast cells by double-stranded RNA: evidence for activation through toll-like receptor 3. J Allergy Clin Immunol. (2004) 114:174–82. doi: 10.1016/j.jaci.2004.03.049, PMID: 15241362

